# Resource pulses shape seasonal and individual variation in the diet of an omnivorous carnivore

**DOI:** 10.1002/ece3.11632

**Published:** 2024-07-04

**Authors:** Alex J. Jensen, Michael Muthersbaugh, Charles R. Ruth, Joseph W. Butfiloski, Jay Cantrell, Jennifer Adams, Lisette Waits, John C. Kilgo, David S. Jachowski

**Affiliations:** ^1^ Department of Forestry and Environmental Conservation Clemson University Clemson South Carolina USA; ^2^ North Carolina Museum of Natural Sciences Raleigh North Carolina USA; ^3^ South Carolina Department of Natural Resources Columbia South Carolina USA; ^4^ Department of Fish and Wildlife Sciences University of Idaho Moscow Idaho USA; ^5^ United States Forest Service Southern Research Station New Ellenton South Carolina USA

**Keywords:** consumer, coyote, deer, intraspecific, optimal foraging, resource availability

## Abstract

Resource pulses are ecologically important phenomenon that occur in most ecosystems globally. Following optimal foraging theory, many consumers switch to pulsatile foods when available, examples of which include fruit mast and vulnerable young prey. Yet how the availability of resource pulses shapes the ecology of predators is still an emerging area of research; and how much individual variation there is in response to pulses is not well understood. We hypothesized that resource pulses would lead to dietary convergence in our population, which we tested by tracking both population‐level and individual coyote diets for 3 years in South Carolina, USA. We (1) described seasonal dietary shifts in relation to resource pulses; (2) compared male and female diets across seasons; and (3) tested this dietary convergence hypothesis by quantifying individual dietary variation both across and within periods when resource pulses were available. We found that pulses of white‐tailed deer fawns and blackberries composed over half of coyote diet in summer, and persimmon fruits were an important component in fall. Male and female coyotes generally had similar diets, but males consumed more deer in fall, perhaps driven by scavenging more. We found support for our dietary convergence hypothesis, where individuals had more similar diets during resource pulses compared to a non‐pulse period. We also found that this convergence happened before peak availability, suggesting a non‐symmetric response to pulse availability. We show that nearly all coyotes eat fawns, suggesting that targeted efforts to remove “fawn killers” would be in vain. Instead, given how quickly coyotes collectively converge on resource pulses, our findings show that resource pulses could potentially be used by managers to alter the behavior of apex predators. More broadly, we open a new line of inquiry into how variation in individual foraging decisions scales up to shape the effects of resource pulses on ecological communities.

## Graphical abstract

Resource pulses are ecologically important; yet the amount of individual, how much individual variation in their use is not well understood. Here, we show that individuals rapidly and collectively converge on resource pulses when available, likely influencing predator–prey interactions.

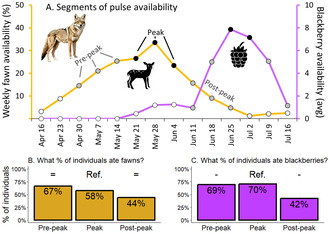

## 1 INTRODUCTION

Food resources are distributed heterogeneously through space‐time and many consumers alter their behavior and diets accordingly (Abrahms et al., 2021; Spitzer et al., 2020). Foods also vary in their spatiotemporal variability—some can be relatively stable in availability through time with high spatial variability, while others are relatively evenly distributed spatially, but with large fluctuations temporally (Wiens, 1976; Yang et al., 2008). According to optimal foraging theory, consumers should behaviorally respond to this spatiotemporal variation when doing so will maximize their net energy gain (MacArthur & Pianka, 1966). Yet these resources can vary at fine scales—both spatially and temporally—so testing optimal foraging theory in this context will require fine‐scale measures of food availability and consumer behavior.

Resource pulses are an example of foods with rapid temporal fluctuations in availability, and they can have disproportionate and cascading impacts on ecosystems (Yang et al., 2008). For example, annual pulses of fruit mast (e.g., acorns and berries) occur in many terrestrial systems, often catalyzing the population growth of consumers which in turn supports population growth of their predators (Ostfeld & Keesing, 2000). The birth of vulnerable young can also be a resource pulse for predators, both because births are often phenologically synchronized (Ims, 1990) and availability to predators can quickly decline as the prey grows (Nelson et al., 2015). Furthermore, many species (particularly omnivores) can respond to multiple resource pulses (Yang et al., 2010), and recent research has highlighted how overlapping vegetative pulses can influence predator response to pulses of vertebrate prey (Deacy et al., 2017). How predators make decisions when multiple resources are available and fluctuating at different timescales has yet to be resolved (Bastille‐Rousseau et al., 2011).

Despite the ecological importance of resource pulses, we know little about individual variability in response to them. In some cases, demographic differences could explain intrapopulation variation in resource tracking—including sex, age, and social status (Masoero et al., 2020; Nilsen et al., 2009; Voigt et al., 2018). But some variation is also likely a product of individual specialization—where a given individual only fills a portion of the niche space used by the whole population (Bolnick et al., 2002). Individual specialization has generally been shown to reduce intraspecific competition (Araújo et al., 2011; Huss et al., 2008), though resource pulses may reduce specialization (and therefore competition) if the population converges around them. It is also unclear how individual variation changes throughout the duration of a given resource pulse. Intuitively, it seems likely that a larger proportion of individuals in the population would converge on the resource pulse during peak availability, but this hypothesis has not been tested to our knowledge. This may be because it is challenging to collect individual data—it requires either observing how individuals change their behavior during the pulse or using molecular approaches to identify the dietary patterns of specific individuals (Seamster et al., 2014).

**FIGURE 1:**
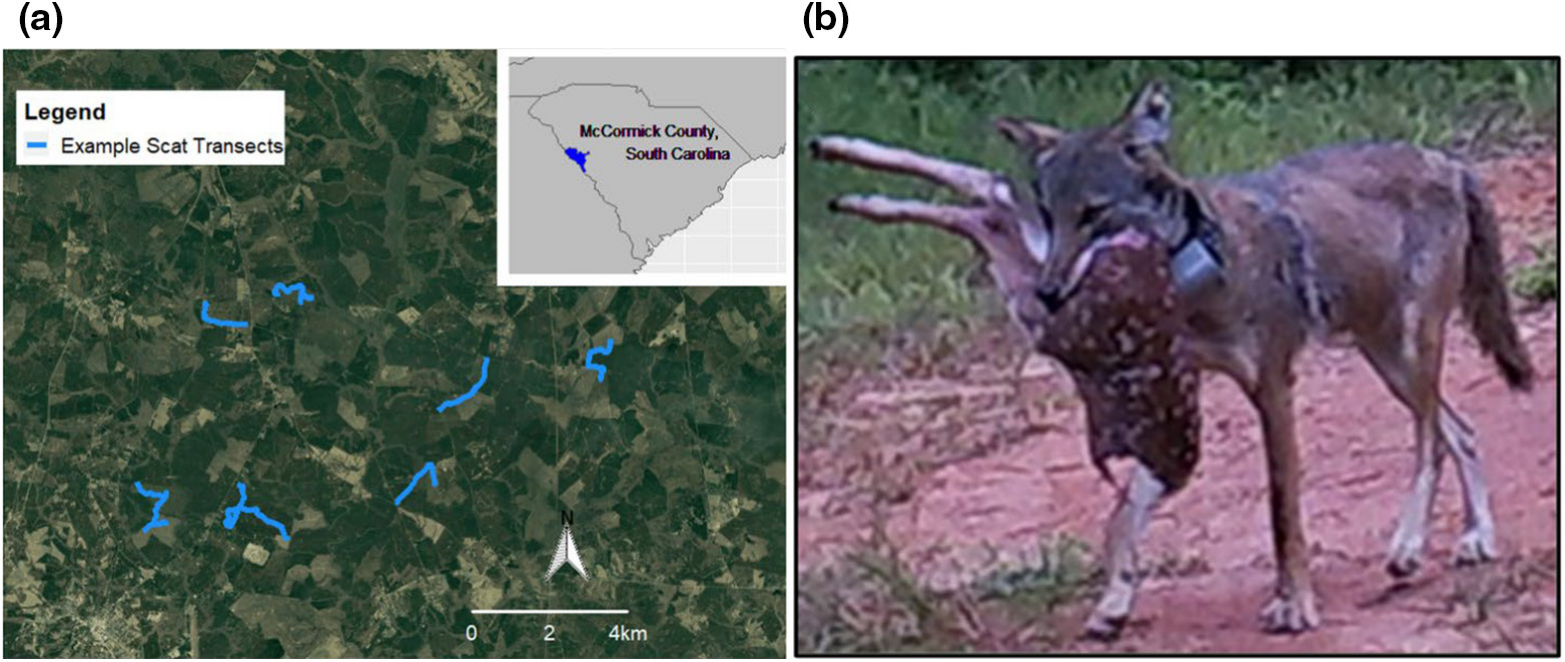
An introduction to our study system. In (a), we show our study area within McCormick County, South Carolina, as well as an example of how our scat transects were distributed across the area. In (b), we show a cropped wildlife camera photo of one of our collared coyotes carring a fawn carcass.

A better understanding of individual variation in response to resource pulses would be particularly useful for apex carnivores, given their important ecological roles; such as their stabilizing effects on systems (Araújo et al., 2011; Rooney et al., 2006) and impacts on game species and livestock (Ripple et al., 2014). In eastern North America, coyotes (*Canis latrans*) are a novel apex predator (Hody & Kays, 2018), where they are having strong top‐down effects within some regions (Kilgo et al., 2010). Indeed, coyotes contribute to white‐tailed deer (*Odocoileus virginianus*) population declines in some regions via fawn predation (Figure 1, Kilgo et al., 2010), leading to wide‐scale persecution of coyotes across eastern North America (Flores, 2016). Yet even intensive coyote removal seems to have marginal effects on fawn survival (Gulsby et al., 2015; Kilgo et al., 2014), and removing resident individuals could increase fawn predation through an influx of immigrants vying for the vacant territory (Knowlton et al., 1999). In addition to fawns, pulsatile fruits are a substantial portion of coyote diet in summer and fall (Cherry et al., 2016; Kelly et al., 2015); yet, we know little about individual variation in fawn predation or fruit consumption. Some demographic groups or individuals might kill more fawns or generally track these pulses more tightly than others. A better understanding of individual variation in convergence around resource pulses could be key to the management of coyotes and other omnivorous apex predators.

Our overall goal was to quantify population level and individual variation in how coyotes responded to fluctuating resources. Our first objective was to describe seasonal diets and estimate how important resource pulses were at the population level. Second, we compared male and female diets across seasons, focusing on four core food items. We hypothesized that males would be more carnivorous, perhaps driven by eating more fawns in summer (Sacks et al., 1999) and scavenging more than females (Ruprecht et al., 2021). Our third objective was to quantify how resource pulses influence individual dietary variation. Here, we tested a *dietary convergence* hypothesis, whereby we predicted that individuals would have more similar diets during periods when resource pulses were available compared to periods when they were not (Bailey & Moore, 2020). We also hypothesized that this convergence would be stronger for the fawn pulse compared to fruit pulses because of the greater caloric benefits from fawns (Gable et al., 2018). Our last objective was to test this dietary convergence hypothesis again, but within the context of a given resource pulse. We hypothesized that availability would shape convergence, where more individuals would consume summer resource pulses (fawns and blackberries) during peak availability compared to periods of rising and declining availability. Finding evidence for dietary convergence would suggest that there is a threshold of availability, whereby a large proportion of the population switches to a resource pulse (Bailey & Moore, 2020). This knowledge can help managers better understand coyote predation on neonates, and also predation in other systems where omnivorous predators key in on multiple, temporally dynamic resource pulses.

## 2 METHODS

### 2.1 Study area

We studied coyote diets across a 61 km^2^ area in McCormick County, South Carolina (Figure 1). Our study area was representative of the Piedmont physiographic region, with a humid subtropical climate, mild winters (typically 3°C–13°C), and gently rolling hills (120–180 m elevation). Most of the land was covered by loblolly pine (*Pinus taeda*) plantations in various successional stages. There were pastures and fields intermittent throughout the landscape, including food plots for game species planted with clover (*Trifolium* spp.), sunflowers (*Helianthus*), and sorghum (*Sorghum* spp.). Midstory and understory native plants that coyotes might eat included persimmon (*Diospyros virginiana*), blackberry (*Rubus allegheniensis*), and muscadine (*Vitus rotundifolia*). Other potential coyote prey included deer, eastern cottontails (*Sylvilagus floridanus*), small mammals, and birds. Indeed, coyotes are responsible for 60% of fawn mortality in our study area, with most predation occurring during the first 3 weeks of a fawn's life (Muthersbaugh, 2023). Hunting occurred throughout the study area, including deer season from October to December. Human density was generally low (<100 people per km^2^); yet, human‐induced coyote mortality was common—roughly 60% of the coyotes we tracked with GPS collars died from being shot (Appendix 1: Text A1).

### 2.2 Survey design and scat collection

We collected scats during three survey periods throughout the year from summer 2019 through summer 2021 (seven total survey seasons). We chose our survey periods to capture potentially important differences in food availability and coyote life history throughout the year. We sampled from mid‐January through mid‐March (winter) when foods typically associated with warmer months (e.g., fawns, fruits, and juvenile small mammals) are typically not available and coyotes are mating. We sampled from early May through mid‐July (summer) to represent a period when vulnerable fawns and fruits (primarily blackberries) were available. Summer is also a period when energetic needs are high as many adult coyotes are rearing pups. We sampled mid‐October through mid‐December (fall) to capture a period when deer hunters potentially leave carcasses or offal for scavenging and when juvenile coyotes typically disperse.

During each season, we regularly surveyed ~16 km of dirt roads broken up into 7–15 transects distributed across our study area (Figure 1). We chose the general location of each season's transects using GPS data from collared coyotes (Appendix 1: Text A1), which ensured that our surveys were within areas known to be occupied. To do this, we mapped the dirt roads in our study area, and then randomly selected a GPS point from each coyote from the previous month which overlapped with the road layer. Once in the field, we went to each GPS point and selected 1–3 km of road (the transect) that would maximize our chances of detecting scats (i.e., dirt road with bare ground) while still including the selected point.

We surveyed transects every 14 days in winter and fall, and every 4–7 days in summer (when heat and humidity degrade DNA faster). Each time we found a scat with collectable fecal material; we estimated its age in days and assigned a field species ID. We used size, shape, apparent contents, and context clues (i.e., tracks) to assign a species ID (Elbroch, 2003), and included a confidence level of low, medium, or high for our ID (Morin et al., 2016). We also pulled off a pea‐sized amount of fecal material from the exterior and placed it in 1.4 mL of DETs buffer (Stenglein et al., 2010), which preserves DNA. We placed the remainder of the scat in a plastic bag and froze it for later dietary analysis. We removed any scats without collectable fecal material from the transect each survey to ensure that we only collected fresh scats during the next survey.

### 2.3 Species and individual scat identification using genetics

We used the samples we preserved in DETs buffer to conduct species identification (SID) of the scat depositor using polymerase chain reactions (PCR). First, we extracted the DNA from the samples using the Qiagen QIAmp Fast DNA Stool Mini Kit (Qiagen, Valencia, CA, USA) in a laboratory dedicated to low‐quantity DNA sources. We then conducted SID PCR with a carnivore primer multiplex (De Barba et al., 2014), which was designed to amplify the mitochondrial DNA of whichever carnivore species' DNA is present. We used the Applied Biosystems 3130xl ABI capillary machine (ABI) and GeneMapper 6 to analyze the lengths of the amplified DNA fragments and determine the species of origin (sensu Stenglein et al., 2011). We determined that samples with peaks in fluorescent intensity ≥100 only at known coyote fragment lengths were deposited by coyotes.

After completing SID, samples with peaks in fluorescent intensity ≥1000 at known coyote fragment lengths were passed on to individual ID. We used a multiplex of 10 nuclear microsatellite loci (2004, CXX119, CXX173, FH2001, FH2054, FH2088, FH2137, FH2611, FH2670, and FH3725) and two canid‐specific sex ID loci (X‐DB6, YDB7; Seddon, 2005). We then conducted two PCRs on each sample with a 94°C denature, 57°C annealing, and 72°C extension conditions for 48 cycles before assessing loci amplification success using a Microsoft Access database, excluding samples with <40% loci amplification. We repeated PCR with the rest of the samples 1–4 more times, or until there was consensus at ≥7/10 loci (Taberlet et al., 1996). We excluded samples without consensus at ≥7/10 loci or without consensus at the sex loci (not clearly from a male or female). Next, we grouped samples with each other using their genotypes. Using 89 coyote tissue samples collected from our study area (Appendix 1: Text A1), we calculated that sample genotypes needed to match at ≥5 loci and to meet a probability of identity siblings threshold of <0.01 as evidence that they came from the same individual using GenAlEx 6.5 (Peakall & Smouse, 2006). When a sample had one allele that did not appear in other samples in that group (*n* = 16 samples), we considered it a match if all the following criteria were met:

1. The sample matched the group's reference sample at ≥ seven loci.
2. The sample's sex (male or female) matched the group's sex.
3. It was collected near other samples in that group (based on GPS coordinates).

### 2.4 Dietary analysis from scats

We quantified coyote diet by isolating the solid material in each coyote scat and identifying it visually. Although genetic approaches to identify food items are becoming more common (i.e., metabarcoding; De Sousa et al., 2019), we elected to use visual identification for two reasons. First, our objectives focused on a core group of easy to visually identify dietary items. Second, we needed to be able to differentiate age classes of deer which, to the best of our knowledge, is not possible via genetic approaches. We dried the frozen scats in an oven at 85° C for 48 h, and then washed them on a stack of five progressively fine metal sieves (6.3 mm, 2 mm, 500 μm, 63 μm, and 1.25 μm), which isolated the solid material. We then air dried the samples for ≥72 h, and then separated the contents into material type (hair, bones, feather, vegetation, insect, or other). We used a combination of technical manuals on mammal hair (Moore et al., 1997; Teerink, 2003), hair reference slides, and online sources to identify the contents of each sample.

We quantified the amount of the following 11 categories in each sample: deer, wild pig, rabbit, small mammal, other mammal (i.e., armadillo, carnivore, and unknown mammal), bird, insect, blackberry, persimmon, other fruit and vegetation (i.e., apple, cherry, and grass/leaves/pine needles/bark), and other (i.e., anthropogenic and unknown). To differentiate fawns from adult deer during summer, we measured the width of five hairs from three <6‐week‐old fawns and three adults from our field site (Ward et al., 2018). We determined that hairs <70 μm were likely from fawns, hairs >90 μm were likely from adults, and hairs 70–90 μm were ambiguous. Therefore, for each sample with deer hair, we measured three randomly selected deer hairs and used the average width to classify the sample as either fawn, adult, or ambiguous. In addition, if a sample from summer was classified as ambiguous based on hair width but we found small hooves in the sample, we reclassified that sample as fawn.

After identification, we estimated the relative amount of the sample made up by each category on a scale of 0–5, where zero was not present, one was <2% (trace), two was 2%–25%, three was 26%–50%, four was 51%–75%, and five was 76%–100% (Prugh, 2005). We converted scores of 1–0 for all food items (Prugh, 2005), and also scores of 2–0 for grass/leaves/pine needles/bark since sometimes these items are collected inadvertently. Because it can be beneficial to compare multiple metrics of carnivore diet when possible (Bojarska & Selva, 2012), we also corrected for digestibility using values from side‐striped jackal feeding trials (*Lupellela adusta*; Atkinson et al., 2002), reported in Loveridge and Macdonald (2003). We multiplied each food item's estimated relative amount (0–5) by the appropriate correction factor, using the medium mammal correction factor for deer and wild pigs because Atkinson et al. (2002) did not include ungulates in their feeding trials.

### 2.5 Objective one: Seasonal patterns in coyote diets

We calculated the % weighted occurrence by summing all the scaled (0–5) or digestion‐corrected (0–31.5) occurrences for a given food item during each season and divided by the total sum of the occurrences for all food items in that season (Prugh, 2005), where *X* is whether or not food item *i* is present in sample *s* (0 or 1) and *Y* is the scaled or digestion‐corrected amount: % weighted occurrence of food item *i* = $\frac{\sum_{s}^{S}X_{\text{is}}Y_{\text{is}}}{\sum_{i}^{I}\mathrm{XY}.}$

We calculated the proportion of each season's diet consisting of resource pulses: fruit (i.e., not grass) and fawns.

### 2.6 Objective two: Comparing male and female coyote diets

**FIGURE 2:**
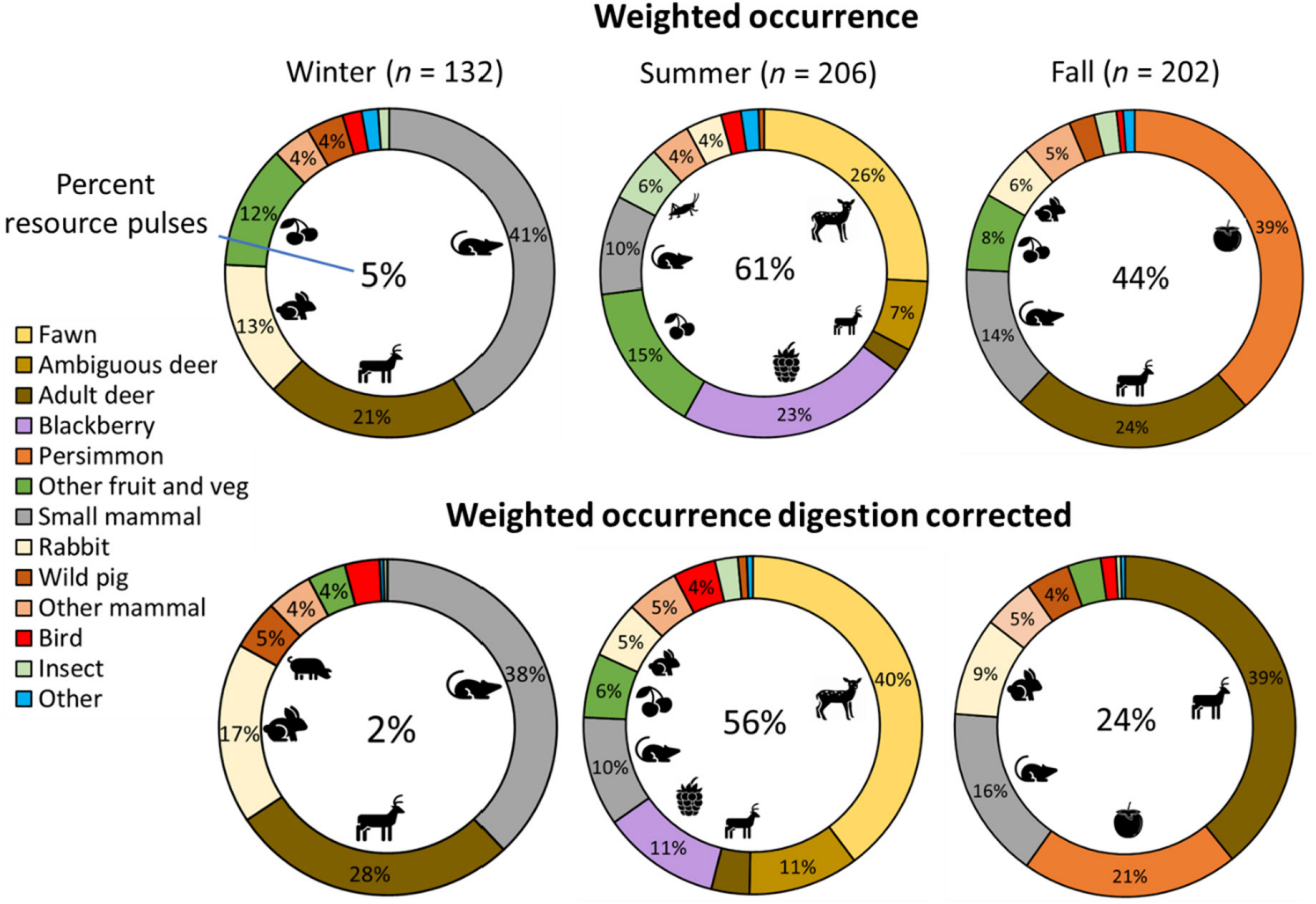
Seasonal coyote diets in South Carolina, USA. We calculated diets using weighted occurrence (top row) and weighted occurrence corrected for digestion (bottom row). In the middle of each circle, we show the percent of the diet consisting of resource pulses (fruit or fawns).

We evaluated support for our hypothesis that there would be dietary differences between male and female coyotes using the subset of our scat samples that were assigned a sex during our genetic analysis (*n* = 333). We binned the data by season, and then compared the sexes within each of the four most consumed food categories: deer, small mammals, rabbits, and vegetation (83%–91% of diets; Figure 2). We fit a single generalized linear mixed model with a Poisson distribution and a random intercept effect of individual for each food item‐season combination (12 models). We considered differences to be significant if 95% confidence intervals did not overlap zero.

### 2.7 Objective three: Comparing dietary convergence across seasons

We evaluated support for our hypothesis that resource pulses would influence dietary variation among individuals by quantifying dietary similarity among individuals in our population during four distinct 6‐week periods. To test our dietary convergence hypothesis, we centered three of these periods around peak availability of temporally discrete resource pulses (fawns, blackberries, and persimmons Figure 2), while the fourth was during winter when resource pulses were largely not available. Our general hypothesis was that resource pulses would lead to dietary convergence in the population, such that, despite a greater diversity of food available in the warmer months (Figure 2), individual diets would be more similar to each other compared with during winter period. We also hypothesized that the type of pulse could mediate how strongly coyotes converge. For example, fawns are much more calorically beneficial than fruit (per unit; Gable et al., 2018) and so we predicted that individuals would be more likely to converge their diets during pulses of fawn availability compared to fruit.

For fawns and blackberries, we tracked which weeks best represented these pulses. For fawns, we used a concurrent study of fawn mortality (Muthersbaugh, 2023), which required capture of white‐tailed deer does in January–April of 2019, 2020, and 2021 using dart guns and rocket nets. We fit each doe with a GPS collar and vaginal implant transmitter (VIT). When the does gave birth (sometime April–July), the VIT would alert us. We would then track to the does location, locate the newborn fawns, and place expandable GPS collars on the fawns. If the fawn did not move for 4–6 h, the GPS collar would send a mortality alert, which we would investigate. To quantify fawn availability, we counted the average number of ≤3‐week‐old fawns each week during each subsequent spring and summer. We focused on these young fawns because we found that weekly changes in population‐level coyote diets most closely followed ≤3‐week‐old fawns and other studies have shown that coyote predation is minimal once a fawn reaches 6 weeks of age (Kilgo et al., 2014; Nelson et al., 2015). Using this weekly availability data, we determined that peak fawn availability was from the 6‐week period of May 4 through June 14.

**FIGURE A1:**
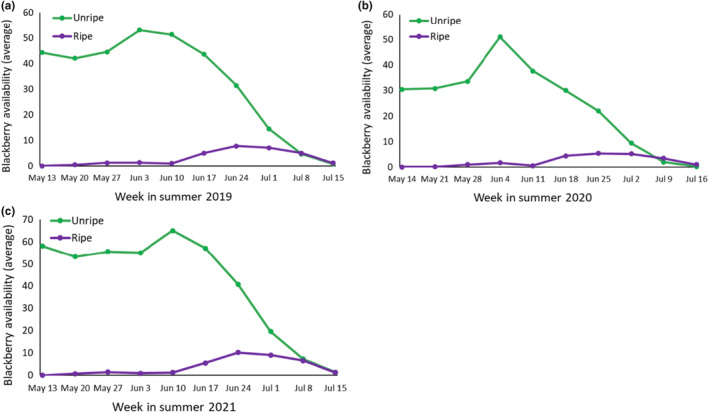
Weekly summer unripe and ripe blackberry availability data in South Carolina. We only counted blackberries in 2020 and 2021, so the weekly counts for 2019 are the average for each week from the following 2 years.

For blackberries, we established plots along dirt roads in our study area, in which we counted the number of ripe blackberries each week. We established four plots per site, and each site was associated with an existing wildlife camera in a 1 km^2^ array (Saldo et al., 2023). We made sure to stratify our sites each year across various forest successional stages (early, middle, and late successional forest) because ripening timing could depend on the amount of sunlight blackberries get. We chose plot locations by walking along the side of the road away from the camera until encountering a blackberry bush. We then set up a 3 × 1 m plot within which we counted the number of ripe blackberries over time. We counted blackberries at four sites in 2020 and eight sites in 2021, and then calculated the average number of ripe blackberries each week (Appendix 1: Text A2; Appendix 3: Figure A1). Peak blackberry availability was during the 6‐week period of June 8 through July 19.

We used two different strategies to determine which weeks to use to represent the persimmon pulse and winter period. We were unable to obtain weekly availability data for persimmons, so we used the 6 weeks of greatest consumption (the weeks of October 8 through November 12), assuming that consumption tracked availability without a temporal lag (like we found was the case for blackberries). For winter period when we observed little dietary evidence of coyotes consuming resource pulses, we randomly selected a period of six consecutive weeks, which was the weeks of January 23 through February 27.

We then calculated similarity between individual diets during each period and compared means statistically. To ensure independence among samples, we randomly removed samples which were from the same individual and collected on the same day on the same transect if we estimated their ages to be within 1 day of each other (assuming we could err our age estimation by 1 day). We excluded these samples from all further analyses. Similar to our previous objective, we only considered variation across the four core food categories (deer, vegetation, small mammals, and rabbits). We calculated the percent weighted occurrence of each of these food categories for each individual during each 6‐week period. We then used these values to calculate pairwise Bray–Curtis dissimilarity between each individual and every other individual using the vegan package (Dixon, 2003). This generated a matrix of dissimilarity scores, where values closer to one indicate less similarity and values closer to zero indicate more similarity. We then compared these values across periods using a Kruskal–Wallis test (Kruskal & Wallis, 1952), followed by a post hoc Dunn test with a Bonferroni correction (Dunn, 1964).

### 2.8 Objective four: Individual convergence during summer resource pulses

We further evaluated support for our dietary convergence hypothesis by comparing how individuals responded to changes in resource availability within a given resource pulse. Using our weekly fawn and blackberry availability data, we identified weeks which corresponded with pre‐peak, peak, and post‐peak segments of each resource pulse (Figure 6). We started by choosing the peak weeks of availability, which for fawns was a 3‐week period from May 18 to June 7. We then used the 3 weeks prior to (April 27–May 17) and after these weeks (June 8–June 28) as the pre‐peak and post‐peak weeks, respectively. The blackberry pulse was shorter and seemed to have a 2‐week peak duration (June 22–July 5; Figure 6), so we used the 2‐week periods of June 8–21 and July 6–Jul 19 for pre‐ and post‐peak, respectively.

To test our prediction that the proportion of individuals that consumed each pulse would be greater during the peak segment compared to pre‐ or post‐peak segments, we quantified individual consumption of each resource pulse during each segment of the pulse. To do this, we collapsed data from each individual during each pulse segment to a 1 or 0 (1 if they consumed any of the pulse) because we were interested in testing for variation across individuals rather than within individuals. We then fit mixed logistic regressions where the response was binary (1 or 0), pulse segment was a fixed effect, individual was a random effect, and the model was weighted by the number of scats. We were also interested in whether individuals ate more of the pulsatile food during peak availability (i.e., variation within individuals), so we fit a similar model where the response variable was individual‐weighted occurrence during each segment. Here, we fit a generalized linear (Poisson distributed) mixed model with pulse‐segment as a fixed effect, individual ID as a random effect, and weighted by sample size.

## 3 RESULTS

We collected 821 total scats from 2019 to 2021, of which 540 (66%) were genetically confirmed to be from coyotes (Appendix 2: Table A1). Most of the remaining samples were bobcat (15%) and unknown (species ID failed; 12%), with few gray fox or red fox (3%; Appendix 2: Table A2). We identified coyote scats correctly in the field 73% of the time and identified bobcat scats correctly 78% of the time (Appendix 2: Table A2).

### 3.1 Objective one: Seasonal patterns in coyote diets

We collected 132 coyote scats in winter (mean = 66 per season), 206 in summer (mean = 69), and 202 in fall (mean = 101). The overall weighted occurrence of foods in coyote diets was 26% deer, 18% small mammal, 15% persimmon, 11% other fruit and vegetation, 9% blackberry, 6% rabbit, 4% other mammal, 3% insect, 2% wild pig, 1% bird, 1% other, and <1% anthropogenic. Other than deer (which were consistently important), coyote diets varied substantially across seasons (Figure 2)—small mammals were the most consumed food in winter, fawns and blackberries were the most consumed in summer, and persimmons were a major component in fall (Figure 2). Indeed, resource pulses (fawns and blackberries) were over 50% of coyote diet in summer and 25%–50% during fall (persimmons; Figure 2). During summer, fawns made up 26% of coyote diets, deer of ambiguous age were 7%, and adult deer were 2%. Correcting for digestion made mammals more important and other food items (i.e., fruit) less important in coyote diets (Figure 2). For example, in summer, vegetation fell from 38% of coyote diet to 17% when correcting for digestion. Similarly, the importance of persimmons and deer reversed when correcting for digestion in fall.

### 3.2 Objective two: Comparing male and female coyote diets

**FIGURE 3:**
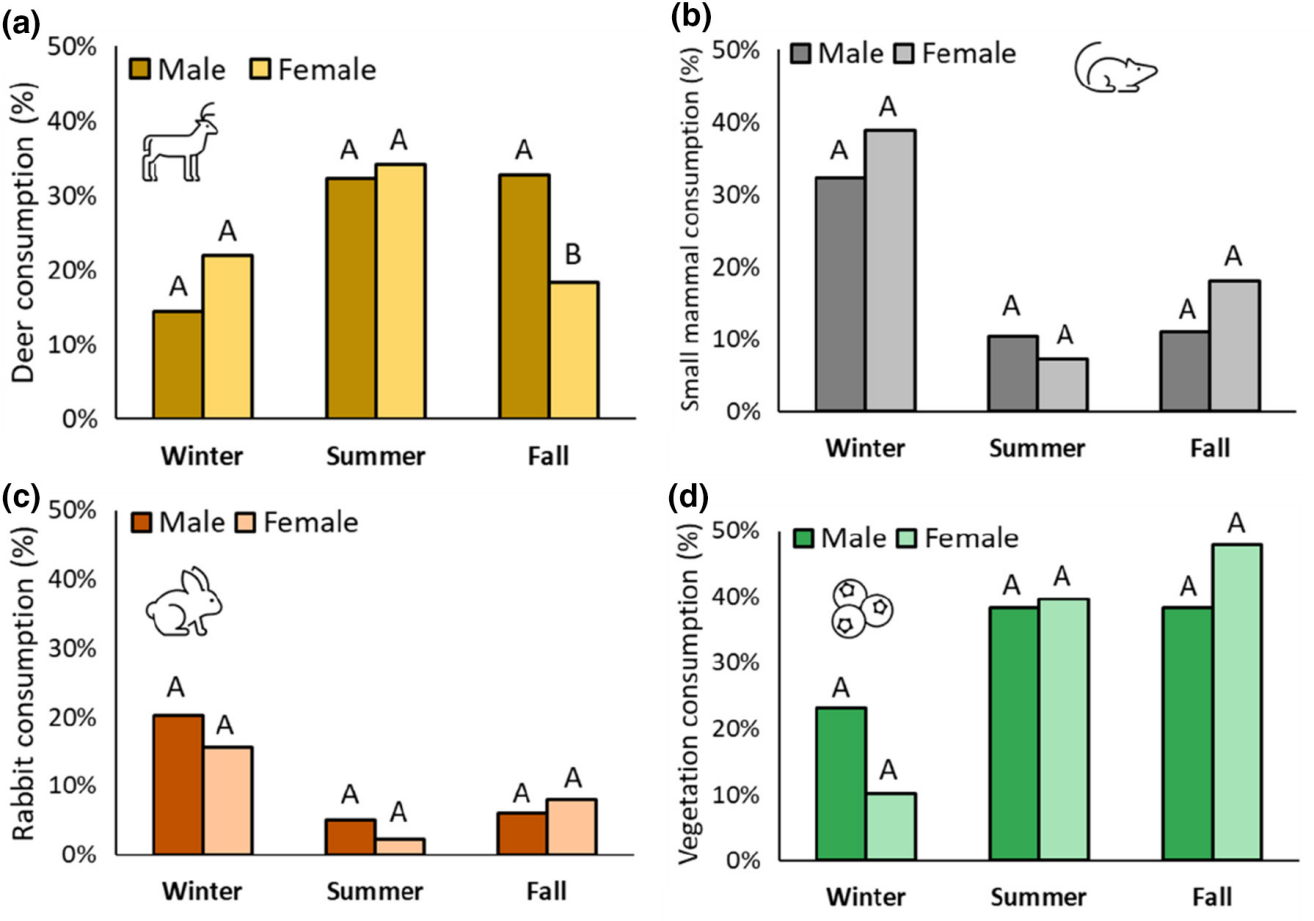
Comparisons of (a) deer, (b) small mammal, (c) rabbit, and (d) vegetation consumption (weighted occurrence) by male and female coyotes in South Carolina. All samples used in this analysis were from genetically individually identified scats, which is how we know the sex of the coyote. Statistical comparisons were between sexes within seasons, where different letters indicate significant differences.

We individually identified 322 coyote scats across all seasons, with nearly equal representation across sexes (166 male and 156 female). Our hypothesis that males would be more carnivorous than females was generally not supported, as they had statistically similar diets in 11/12 of the season food item combinations we examined (Figure 3). The exception being that we found males proportionally ate 15% more deer than females in fall (estimate [95% confidence interval] = 0.98 [0.15: 1.81]).

### 3.3 Objective three: Comparing dietary convergence across seasons

**FIGURE 4:**
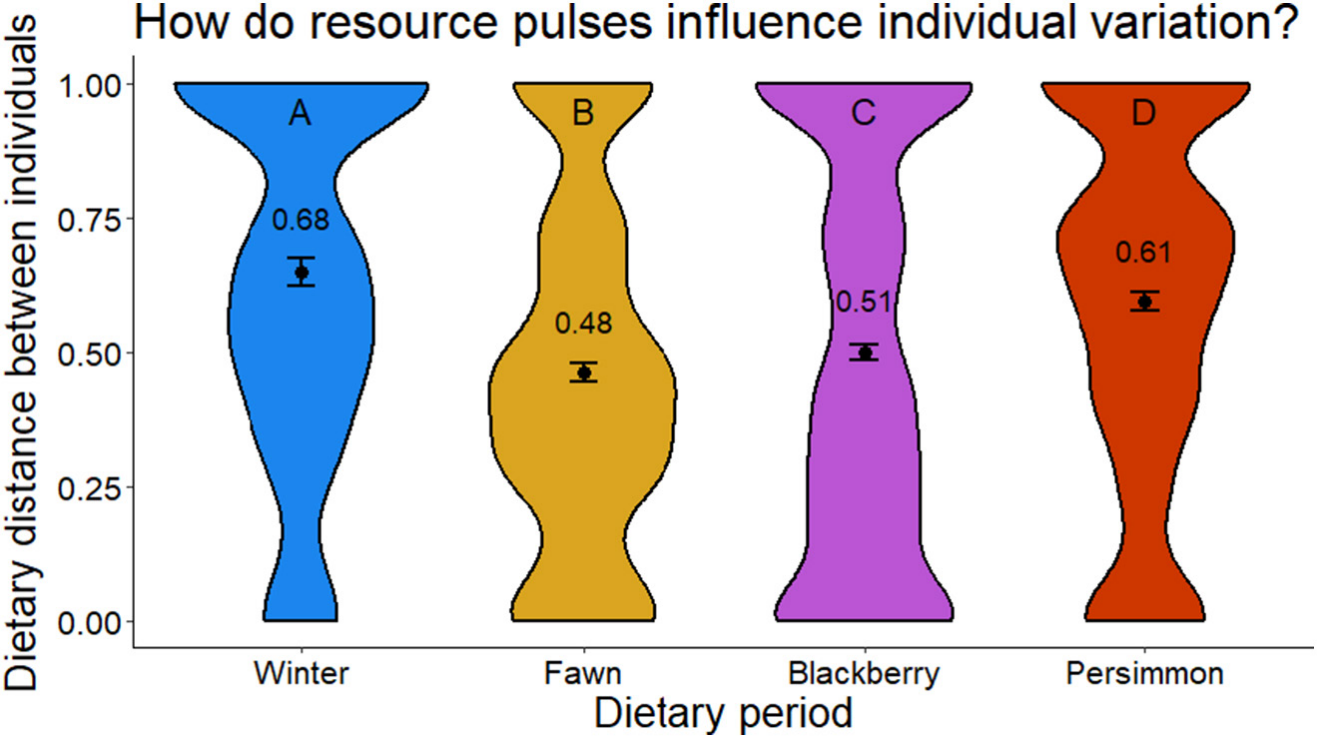
The distribution (violin plots) of dietary similarity values between pairs of coyotes during four distinct 6‐week periods. The fawn, blackberry, and persimmon periods are all centered around when we estimate peak availability to be, while winter period is a control when resource pulses are largely not available. The black dots indicate the mean value, while the error bars represent 95% confidence intervals around the mean. The letters at the top indicate that each period's mean is significantly different from the others according to the Dunn test (Table A3).

We quantified dietary similarity across 40 individuals during the fawn pulse, 52 individuals during the blackberry pulse, 41 individuals during the persimmon pulse and 26 individuals in winter. Overall, we found support for our hypothesis that resource pulses increased dietary convergence across individuals (Figure 4), where diets were the least similar during winter (0.68), following by during the persimmon pulse (0.61), the blackberry pulse (0.51), and the fawn pulse (0.48). We also found that the mean of each of these periods was significantly different from other periods (Table A3), supporting our hypothesis that the type of resource pulse would mediate the strength of dietary convergence.

### 3.4 Objective four: Individual convergence during summer resource pulses

**FIGURE 5:**
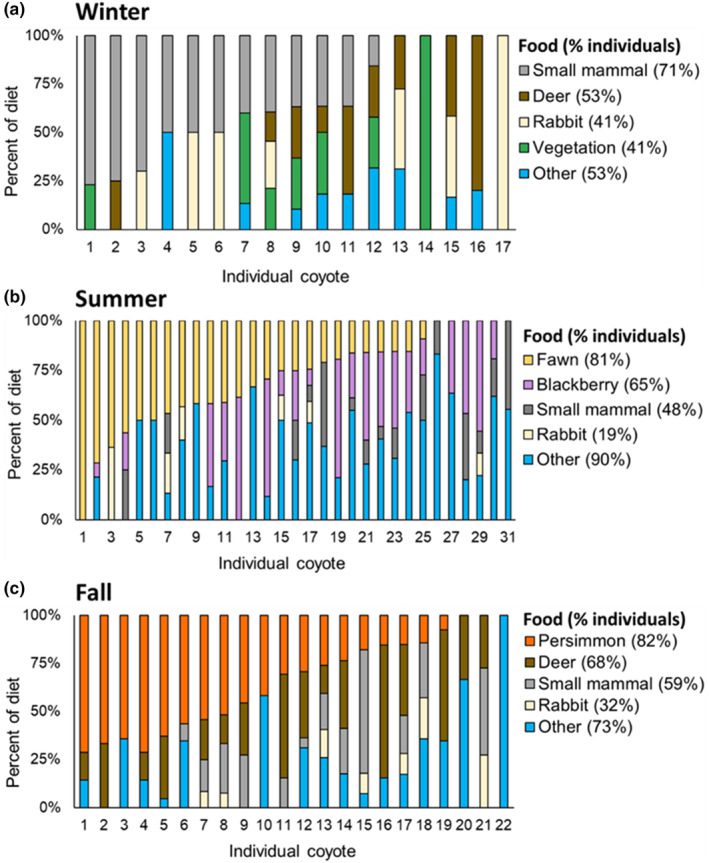
Individual variation in coyote diets across seasons in South Carolina, USA. For each season, we show the top four food categories separately, and then group the remaining foods into the other category. We only show data from individuals from whom we collected at least two scats each season. The percentages indicate the proportion of individuals who consumed that food item.

We collected 146 individually identified scats during our summer surveys over 3 years. During the fawn pulse, we found 91 scats from 56 individuals and during the blackberry pulse, we found 75 scats from 52 individuals. On average, individuals contributed 1.3 scats (range = 1–5). We found that fawns were eaten by 81% of individuals and blackberries were eaten by 65% of individuals (with at least two samples per individual; *n* = 33; Figure 5). Although not in summer, we found a similar pattern in fall where 82% of individuals (*n* = 22) consumed persimmons.

**FIGURE 6:**
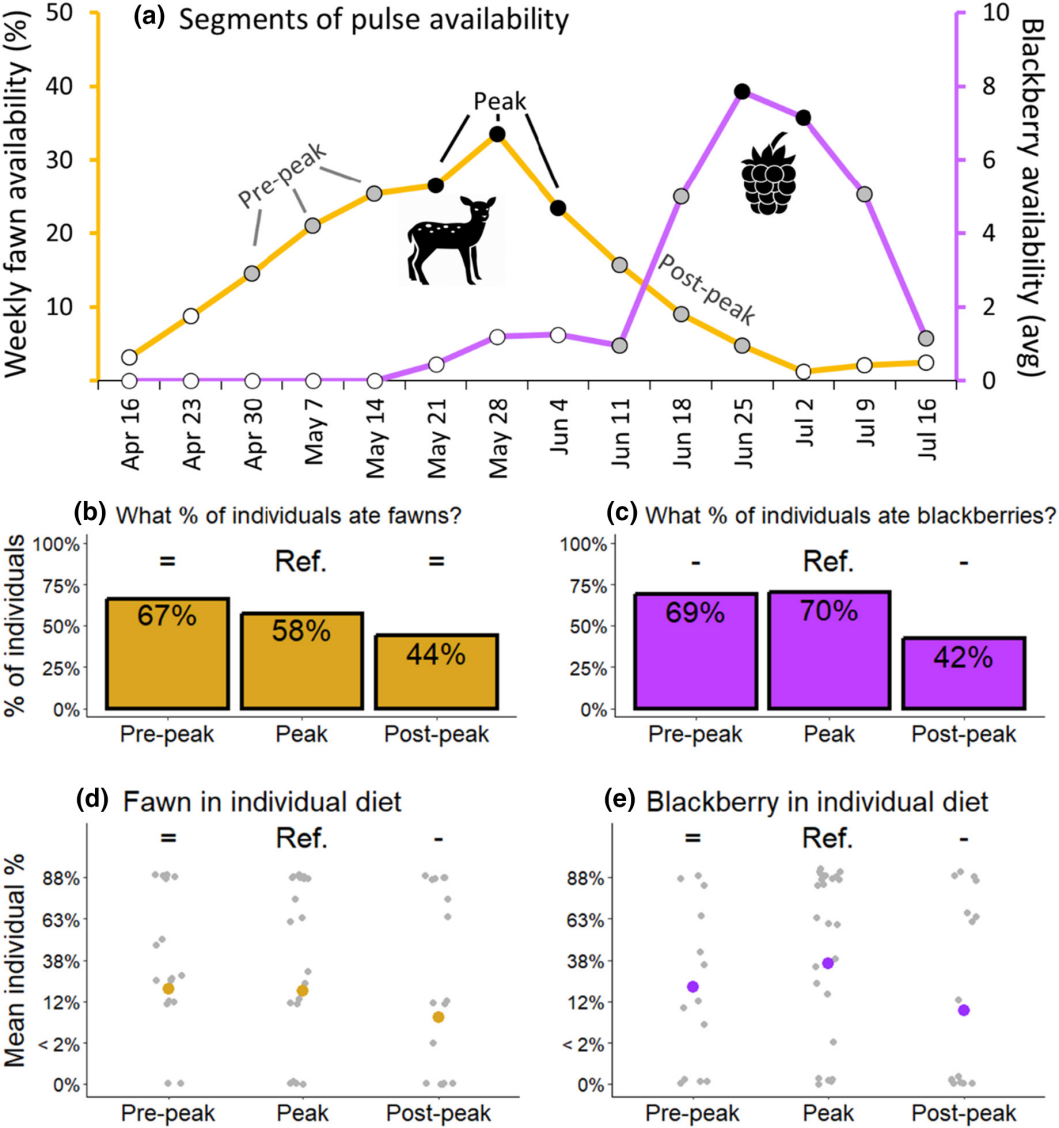
Availability and individual variation in consumption of fawns and blackberries by coyotes in South Carolina, USA. In (a), we show the weekly availability of fawns and blackberries, indicating how we classified our data into pre‐peak, peak, and post‐peak categories. In (b and c), we show the proportion individuals which ate any amount of these pulses. In (d and e), we show the mean percentage of each food, where gray points are individual averages. The letters within the plots denote statistical differences within each plot.

We found partial support for our hypothesis that individual diets would converge the most during peak availability (Figure 6). For coyote consumption of fawns, we found a downward trend in the percentage of individuals that consumed fawns, from 67% during pre‐peak to 44% post‐peak, though neither were statistically different than the peak (58%; Figure 6b). There was a similar trend for the *amount* of fawns individuals ate across segments (Figure 6d), but here we found they ate less fawns post‐peak compared to peak (−0.55 [−1.08: −0.03]). For consumption of blackberries, we found that more individuals ate blackberries during peak (70%), compared to pre‐peak (69%, −22.40 [−37.51: −7.28]) and post‐peak (42%, −19.60 [−27.69: −11.50]). We found similar results for the amount of blackberries individuals ate, where coyotes ate less blackberries post‐peak compared to peak availability (−0.44 [−0.86: −0.02]; Figure 6e). Although the percentage of individuals that consumed blackberries were quite similar between peak and pre‐peak, weighting by sample size likely contributed to this significance because removing weighting from the model caused the difference to be insignificant. Indeed, 26% of individuals in the peak segment contributed more than one scat while only 8% (*n* = 1) of individuals contributed more than one scat in the pre‐peak segment.

## 4 DISCUSSION

Recent research has highlighted how populations of the same species can vary in how they respond to resource pulses (Bowersock et al., 2023; Middleton et al., 2018), yet there has been little research on intrapopulation variation in response. We show that resource pulses influence dietary variation at multiple scales—across seasons at the population level and across individuals within seasons. Resource pulses were major components of coyote diet in summer and fall, yet rarely available in winter when coyotes primarily ate staple foods (small mammals and rabbits). Dietary variation across individuals was lower during periods when resource pulses were available (supporting our dietary convergence hypothesis), but similarity among individuals depended on the type of pulsatile resource available. By tracking fine‐scale changes in the availability of summer resource pulses, we showed that individuals generally shifted to consume resource pulses prior to peak availability and consumed them least during the decline. Taken together, our findings show that resource pulses drive dietary convergence—not only broadly across seasons but also around the peak in availability as well.

One potential explanation for intrapopulation variation in diet is differences in nutritional demands or foraging strategies across demographic groups. For example, a study on lynx in Norway (*Lynx lynx*) showed that females with kittens had higher roe deer (*Capreolus capreolus*) kill rates compared to solitary females, likely linked to increased energetic demands (Nilsen et al., 2009). However, dietary differences between male and female carnivores are typically attributed to sexual size dimorphism (Bauld et al., 2022), where larger males are able to kill larger prey (Magnusdottir et al., 2012; Voigt et al., 2018). Yet we found that male and female coyote diets were quite similar across seasons, perhaps because males in our population are only ~2 kg heavier than females (on average). Indeed, both coyote sexes consumed resource pulses in similar amounts, which did not support our prediction that males would consume more fawns (Sacks et al., 1999).

The only difference between males and females we found was in fall, when males were either killing or scavenging more deer than females. It is possible that the slightly bigger males are better able to kill yearling or adult deer during this season (Cherry et al., 2016; Chitwood et al., 2014; Youngmann et al., 2022); but given how drastically predation rates on fawns decline as they age (Muthersbaugh, 2023; Nelson et al., 2015) and how important scavenged ungulates are in coyote diets (Prugh & Sivy, 2020), we suspect that scavenging largely drives this difference. Indeed, in Oregon, scavenged ungulates were up to 70% of coyote diets, and resident male coyotes were the primary demographic scavenging at puma kills (Ruprecht et al., 2021). Scavenging from large carnivore kills is risky (Prugh & Sivy, 2020), as is scavenging near development or along roads in our system (where most carrion would likely be), so perhaps male coyotes are less risk averse compared to females. Future studies could test how demography influences risk–reward tradeoffs, particularly in the context of resource pulses.

By comparing diets across individuals, we show that resource pulses facilitate dietary convergence. In particular, individual diets were more similar during periods when resource pulses were available (fawn, blackberry, and persimmon pulses) compared to a control period when they were not (winter). This aligns with our dietary convergence hypothesis, but is still somewhat counterintuitive given the diversity of foods available to coyotes is lowest in winter and greatest in summer (Jensen et al. 2022). We propose two mutually inclusive mechanisms which may explain this pattern. First, although converging on the same resource pulse should increase intraspecific competition (Araújo et al., 2011; Huss et al., 2008), perhaps the benefit to a given converging individual outweighs the benefit of specializing during this period (Bolnick et al., 2003). This is supported by our finding that convergence was greatest during the fawn period, as this resource pulse would have a much greater caloric benefit per unit compared to fruit (Gable et al., 2018). Second, not only is the diversity of available food lower in winter, prey abundance is also typically lower during this period of colder temperatures and reduced productivity (McMeans et al., 2015). This lower availability may thus increase competition and facilitate dietary *divergence* among individuals in the population. Indeed, a field experiment with stream fishes showed that more abundant resource pulses facilitated their use by smaller (less dominant) individuals (Bailey & Moore, 2020). Regardless of the mechanism, these results show that individual variation in diet is dynamic across seasons and likely mediated by the availability of resource pulses.

We also show that dietary convergence can vary during resource pulses themselves. We expected convergence to track availability, but instead we found that convergence was just as high during the period of increasing availability as during peak availability. This rapid convergence across individuals to take advantage of an emerging pulse of a resource could be adaptive, as individuals each try to maximize their benefits from these ephemeral foods given they are likely unaware of when availability will peak (Nathoo et al., 2022). Yet once they detect a decline in availability, perhaps it becomes adaptive to switch off a declining pulse in favor of other foods (Huggler et al., 2023). Indeed, reduced fawn consumption post‐peak is likely partially explained by coyotes switching to blackberries as they become available, as has been observed in bear‐salmon‐berry systems elsewhere (Deacy et al., 2017). We found a similar pattern of rapidly declining consumption of blackberries post‐peak compared to pre‐ and peak availability. Given there was not a subsequent pulsatile resource following blackberries (unlike fawns), we hypothesize that individuals switched to other foods during declining availability due to the relatively short duration (Yang et al., 2010) and relatively minimal caloric benefit of blackberries (Gable et al., 2018). This is likely also true for other pulsatile fruits like pokewood (*Phytolacca decandra*) and muscadines (*Vitis rotundifolia*), which were shown to be an important component of coyote diet in August (a month we did not sample in) by another study in South Carolina (Schrecengost et al., 2008).

Coyote dietary convergence on resource pulses has implications for the management of coyotes and other adaptable predators. Coyotes are particularly effective fawn predators in the southeast (compared to other regions in eastern North America; Kilgo et al., 2019); yet, there was no prior knowledge about how common this behavior is within coyote populations. Past studies on sheep predation showed that breeding coyotes are primarily responsible (Blejwas et al., 2002; Sacks et al., 1999), suggesting that this could also be the case for fawns. Yet here we show that nearly all the coyotes we sampled ate fawns and both sexes ate them a similar amount, such that efforts to remove certain individuals primarily responsible would likely be futile. Instead, it has been suggested that managers could potentially distract coyotes from predating fawns by strategically increasing the availability of natural alternative foods (such as blackberries) while fawns are vulnerable (Cherry et al., 2016). While this hypothesis needs to be tested experimentally, our findings add additional weight to this suggested management action by showing that the same ability to rapidly converge on fawns also facilitates a rapid convergence on a subsequent resource pulse.

These findings are also likely relevant in the context of predator management more broadly as previous studies have generally concluded that supplemental feeding (functionally an artificial resource pulse) did not reduce predation on focal prey species (Conover et al., 2005; Kubasiewicz et al., 2016; Morehouse & Boyce, 2017). Although many factors ultimately influence a consumer's response to resource pulses (Yang et al., 2010), our work suggests that there are not only thresholds of availability which lead to rapid population‐level convergence but also that the degree of convergence depends on the type of resource pulse. Finding these threshholds (and how they vary across different resources) could be useful for managers interested in managing predator behavior.

Future studies could expand on our work here in at least two key ways. First, small individual sample sizes limited our ability to conduct additional comparisons among individuals. At least 10 scats per individual would have been needed to defensibly track their diets across seasons (Prugh et al., 2008) or explicitly quantify individual specialization through measures of niche width (Araújo et al., 2011; Bolnick et al., 2003). Second, additional sources of individual variation could be explained by demographic groups which we did not investigate. For example, territorial status (resident or transient) has been shown to influence how coyotes select habitat in the southeast (Webster et al., 2022), as well as predict ungulate kill rates in Eurasian lynx (*Lynx lynx*; Nilsen et al., 2009). It would be particularly interesting to link individual diet and movement data to see if transient coyotes eat fewer fawns.

In a previous study, we showed that coyote dietary ecology varied widely across their range (Jensen et al. 2022), while here we showed that individual variation was also plastic and dependent on the availability of resource pulses. Indeed, resource pulses were major components of coyote diet when available, likely buffering predation on fawns, as well as small mammals and rabbits to some extent. We also showed that individuals quickly converged around summer resource pulses (prior to peak availability), suggesting an asymmetrical response that is dependent on whether the pulse is on the upswing or downswing, which should be tested in other systems. Thus, variation in the timing and availability of resource pulses is likely one driver of range‐wide variation in coyote diet, with local effects depending on a variety of factors (Yang et al., 2010).

There is increasing recognition that variation within species has important ecological and evolutionary implications (Bolnick et al., 2003; Rollinson et al., 2021). We build on this literature by quantifying how resource pulses influence dietary variation within a population of omnivorous apex predators. Our findings suggest that resource pulses drive dietary convergence in populations, likely indirectly benefitting other prey items during periods when they are available. Although convergence would generally lead to increased intraspecific competition, high resource pulse availability would reduce the influence of intraspecific dominance hierarchies on foraging strategies (Bailey & Moore, 2020). Understanding the thresholds of availability that lead to adaptive convergence will be key for predicting population‐level responses to resource pulses. By tracking the availability and consumption of resource pulses at fine scales, we show that consumers respond rapidly, and therefore, the “optimal” foraging behavior could also change at this same rate. Future studies investigating optimal foraging theory in wild systems should consider how quickly consumers can respond to fluctuations in availability.

## AUTHOR CONTRIBUTIONS

**Alex J. Jensen:** Conceptualization (lead); data curation (lead); formal analysis (lead); investigation (lead); methodology (lead); validation (lead); visualization (lead); writing – original draft (lead). **Michael Muthersbaugh:** Data curation (supporting). **Charles R. Ruth:** Conceptualization (supporting); funding acquisition (equal); project administration (equal). **Joseph W. Butfiloski:** Conceptualization (equal); funding acquisition (equal); project administration (equal). **Jay Cantrell:** Conceptualization (equal); funding acquisition (equal); project administration (equal). **Jennifer Adams:** Data curation (supporting); methodology (supporting); software (equal). **Lisette Waits:** Data curation (supporting); methodology (supporting); project administration (supporting); software (supporting); supervision (supporting); writing – review and editing (supporting). **John C. Kilgo:** Conceptualization (supporting); funding acquisition (supporting); project administration (supporting); writing – review and editing (supporting). **David S. Jachowski:** Conceptualization (supporting); funding acquisition (lead); investigation (supporting); methodology (supporting); project administration (lead); supervision (lead); writing – review and editing (lead).

## FUNDING INFORMATION

Funding was provided by the South Carolina Department of Natural Resources, grant number 2012805.

## CONFLICT OF INTEREST STATEMENT

The authors declare no competing interests.

## Data Availability

Our data and code are available on Figshare at the following link: https://figshare.com/s/cc087eea3d37093a236d

## APPENDIX 1

### 1.1 Text A1: Description of methods used to capture and collect tissue samples from coyotes in McCormick County, South Carolina

We captured coyotes using Minnesota MB 550 foothold traps (Minnesota Trapline Products, Pennock, MN) set on dirt roads that had signs of coyote activity (i.e., tracks and scat) during December 2018, January–March 2020, and January 2021. We immobilized coyotes using a catchpole and electrical tape wrapped around their muzzle and ankles. We collected a tissue sample from their ear. The average handling time was 25 min. All capture and handling procedures were permitted by Clemson University IACUC permit no. AUP 2018‐031 and USDA Forest Service permit no. USFS 2018‐031. We programmed our GPS collars to collect data for ~15 months before dropping off automatically. The default fix rate was 7 h, which generally changed to 30 min during three 3‐week windows from February 8 to 28, April 19 to July 7, and October 10 – October 31.

### 1.2 Text A2: Description of methods used to estimate blackberry availability during summer in McCormick County, South Carolina

In 2020, we classified our each of our camera sites (Saldo et al., 2023) into four classes of vegetation (mature pines, immature pines, clear‐cut, mixed pine, and hardwood), then randomly chose one site from each class to count blackberries at. Once at the site, we set up four 3 x 1 m plots, in which we counted blackberries each week (Figure 2). We chose the plot locations by walking along the road for up to 50 m or until we saw a (usually unripe) blackberry, then sticking four flags in the ground to delineate the plot. At each plot, we used a hand counter to tally the number of not‐ripe (green and/or red) and ripe (fully dark purple) blackberries within each plot. In order to create a weekly index from this data, we averaged the counts of the four plots from each site, then averaged those four numbers across the four sites. In 2021, we used eight total sites (two from each habitat class) and counted a group of four every other week. We then interpolated the missing weeks (except the first and last week) for each site by averaging the counts from the week prior and the following week. We averaged weekly counts across all sites to create the weekly index for 2021. Since we did not count blackberries in 2019, we combined our 2020 and 2021 count data to represent 2019. We first examined the blackberry data from 2020 and 2021 to ensure they were similar; we found that unripe blackberries peaked the first week of June in 2020 and the second week of June in 2021, but ripe blackberries peaked the fourth week of June both years (Figure A1). We deemed these trends similar enough to combine, so we averaged the counts for each week across years.

## APPENDIX 2

**TABLE A1:** Sample sizes for our analyses of coyote diet in South Carolina, USA. All the scats in the population column are from coyotes. We found scats in multiple seasons from some individuals, which is why the number of individuals total is less than the sum of the above columns.

Seasons	Population scat samples	Individually ID scat samples	Number of unique individuals
Winter	132	66	37
Summer	206	146	75
Fall	202	110	58
Total	540	322	117

**TABLE A2:** Summary of carnivore scat identification in South Carolina.

Coyote		Predicted (field identification)			Accuracy 73% ^(a+d/(a+b+c+d))^	
		Not a coyote (negative)	Is a coyote (positive)	Total		
Actual (genetic species identification)	Not a coyote (negative)	45^a^	96^b^	141	True negative rate 32%^(a/(a+b))^	False positive rate 68% ^(b/(a+b))^
	Is a coyote (positive)	90^c^	450^d^	540	False negative rate 17% ^(c/(c+d))^	True positive rate 83% ^(d/(c+d))^
	Unclear (failed and multispecies)	28	112	140		
	Total	163	658	821		

Bobcat		Predicted (field identification)			Accuracy 78%	
		Not a bobcat (negative)	Is a bobcat (positive)	Total		
Actual (genetic species identification)	Not a bobcat (negative)	499	59	558	True negative rate 89%	False positive rate 11%
	Is a bobcat (positive)	93	31	124	False negative rate 75%	True positive rate 24%
	Unclear (failed and multispecies)	118	22	140		
	Total	710	111	821		

*Note*: We compare our field species identification with the species identification using genetics (DNA fragment analysis). (a) is a true negative, (b) is a false positive, (c) is a false negative, and (d) is a true positive. We were more likely to falsely identify a coyote scat as a bobcat in the field—65% of scats we identified as bobcat in the field were from coyotes, and 17% of scats we identified as coyote in the field were from bobcats. We based our table layout on Morin et al., 2016: Bias in carnivore diet analysis resulting from misclassification of predator scats based on field identification.

**TABLE A3:** Model results from our Dunn test (nonparametric post hoc mean comparison) on dietary similarity among coyotes during different periods in South Carolina, USA. Below the diagonal, we show *p* values, and above, we show Z values.

Periods	Winter	Fawn	Blackberry	Persimmon
Winter	NA	*Z* = −10.6	*Z* = −9.3	*Z* = −3.3
Fawn	*p* = <.001	NA	*Z* = 2.9	*Z* = −9.5
Blackberry	*p* = <.001	*p* = .01	NA	*Z* = −8.0
Persimmon	*p* = .003	*p* = <.001	*p* = <.001	NA
